# Letter from a Distinguished Medical Gentleman

**Published:** 1871-05

**Authors:** 


### LETTER FROM A DISTINGUISHED MEDICAL GENTLEMAN.

Drs. Powell & Goldsmith:—The last number of your valuable Companion has fallen into my hands. I am forced to the conclusion, from a close examination of its editorials, selections and extracts, that it is superior to any other Medical Journal in *this* country. You will please forward me the remaining back numbers, and I will remit you the subscription price.

My intention was to have attended the last annual meeting of the Georgia Association, but since I have read the proceedings of that body, I am glad that my previous engagements prevented. I sent a short communication to be read to that organization, which was not done, and I am pleased that my friends for certain reasons withheld it, which has met my entire approbation.

While I regret to see the unpleasant circumstances which occurred at the meeting of the Association, I most cheerfully and heartily sanction the protest signed by a number of the gentlemen who were present; and I am compelled to conclude that the unconstitutional and illiberal proceedings of the majority, who, we are informed, are new members, will eventually break up the Association, and widen the breach.

I remember to have read, a few years ago, a memorial to the Legislature by the Faculty of the Atlanta Medical College, which I then regarded as a gross outrage upon the profession of Georgia; and I had hoped that the charges alledged would have been retracted, and unanimity and good feeling been restored among all parties ; but the preamble and resolution offered by Dr. Logan at the last Georgia Medical Association not only fails to retract any of those charges, but leaves them remaining in full force, and adds additional insult to the whole profession, which must culminate and progress until the "feeling will not be confined to Atlanta, but will be wide-spread in the State ;" and not only in this State, but throughout the United States ; from the fact that whatever affects the great cardinal principles of the profession in one section, operates with equal force upon all the rest. From all I have heard from various sources, I suppose that there might have been some little personal feeling existing among some of the gentlemen connected with the Association and you gentlemen in Atlanta ; but I understand that while this may be true, that none of the parties opposed to the illegalities or irregularities of the Atlanta Medical College, have at any time introduced their *personal feelings* into the Association, and "ought never to have been," but, it seems to have been done for the first time by Dr. Logan's resolutions. To my mind, it is clear that you gentlemen are not responsible for the new issue he makes with the profession.

While I am satisfied that your long experience forestalls any opinion from me as to advice, yet I will venture to suggest that, as your action has been sustained by the Georgia and American Medical Associations, I can see but little to be done now by you gentlemen, as individuals, but to stand firm, as the whole issue involved must be met and managed by all who composed the Georgia Medical Association, for the last three or four sessions. If the charges preferred in the memorial be *true*, and all the actions of the Association in regard to it be *false*, then the profession of the State is disgraced, and none worthy of confidence but the Atlanta Medical College and its Faculty. If, however, the last proceedings of the Georgia Medical Association should prove to be unconstitutional and unprecedented, then Dr. Logan's resolution does not rescind the previous action of the Georgia Medical Association in reference to the Atlanta Medical College, its Faculty and its students, but will be considered in full force by the profession, and the College must be in a worse condition than before, as medical gentlemen, who are the preceptors, will not permit their students to attend an institution of such doubtful reputation with almost the entire profession.

And in conclusion, gentlemen, let me now state, that this controversy is now no longer a *personal one*—but between the profession and the Atlanta Medical College, and I advise you to stick to your integrity, battle for the truth—never suffer *personalities* to be dragged in as the means of diverting the minds of the profession from the main issues involved. The people are deeply interested—they regard the institution as the property of the State, and the people will expect and demand at the hands of the Trustees as a duty they owe the whole country to place the Institution in that position as will secure its prosperity and success, and close this controversy.

It is very evident it can never prosper so long as the Trustees pay no respect to the will of the profession, and its Professors seek to degrade all but themselves. As we understand it in our town, the controversy is now with Dr. Logan and his party and the Georgia Medical Association up to its last session of 1871.

